# Working memory during spontaneous migraine attacks: an fMRI study

**DOI:** 10.1007/s10072-023-07120-0

**Published:** 2023-10-17

**Authors:** Amparo Ruiz-Tagle, Patrícia Figueiredo, Joana Pinto, Pedro Vilela, Isabel Pavão Martins, Raquel Gil-Gouveia

**Affiliations:** 1 Instituto Superior Técnico, Universidade de Lisboa, ISR-Lisboa/LARSyS and Department of Bioengineering, Lisbon, Portugal; 2 Centro de Estudos Egas Moniz, Faculty of Medicine, Universidade de Lisboa, and Hospital de Santa Maria, CHULN, Lisbon, Portugal; 3 Institute of Biomedical Engineering, Department of Engineering Science, University of Oxford, Oxford, UK; 4 Serviço de Neurradiologia, Hospital da Luz, Lisbon, Portugal; 5 Headache Center, Serviço de Neurologia, Hospital da Luz, Lisbon, Portugal; 6 Center for Interdisciplinary Research in Health, Universidade Católica Portuguesa, Lisboa, Portugal

**Keywords:** Spontaneous migraine attack, fMRI, Cognition, Test-retest, N-back

## Abstract

**Objective:**

To investigate the neural correlates of working memory during a spontaneous migraine attack compared to the interictal phase, using functional magnetic resonance imaging (fMRI).

**Background:**

Cognitive disturbances are commonly observed during migraine attacks, particularly in the headache phase. However, the neural basis of these changes remains unknown.

**Methods:**

In a fMRI within-subject test-retest design study, eleven women (32 years of age, average) with episodic migraine were evaluated twice, first during a spontaneous migraine attack, and again in a pain-free period. Each session consisted in a cognitive assessment and fMRI while performing a working memory task (N-back).

**Results:**

Cognitive test scores were lower during the ictal session than in the pain-free session. Regions typically associated with working memory were activated during the N-back task in both sessions. A voxel wise between session comparison showed significantly greater activation in the left frontal pole and orbitofrontal cortex during the attack relative to the interictal phase.

**Conclusion:**

Migraine patients exhibited greater activation of the left frontal pole and orbitofrontal cortex while executing a verbal working memory task during a spontaneous migraine attack when compared to the interictal state. Given the association of these regions with pain processing and inhibitory control, these findings suggest that patients recruit inhibitory areas to accomplish the cognitive task during migraine attacks, a neural signature of their cognitive difficulties.

## Introduction

Migraine is a prevalent neurological disorder that affects approximately an 11.6% of the global population [1], and according to the Global of Burden Disease study 2019, it is the second cause of neurological disability in young and middle-aged adults [2]. Migraine is characterized by a cyclical and episodic pattern of recurrent attacks (ictal phase) of severe headaches and other associated symptoms that include reversible cognitive disturbances [3, 4]. These cognitive difficulties are reported by almost 40% of patients and consist in attention and word retrieval difficulties, executive dysfunction with impairments in multitasking, inhibitory control, attention shifting and working memory [5, 6]. To date, few studies have objectively analysed the nature of cognitive disturbances during spontaneous migraine attacks. Two of such studies used a within-subject design, wherein migraine participants were assessed twice, during the ictal phase and during a pain-free period (interictal phase) and found a consistent decline in performance during the ictal phase, which was significant for cognitive screening tests and executive function tasks [7, 8]. Although pain may contribute to these difficulties, it cannot completely explain the cognitive decline since cognitive complaints often begin before the onset of headache, in the preictal phase [9].

Studies using resting-state functional Magnetic Resonance Imaging (fMRI) have shed light on the complex interaction between cognitive and pain processing areas during pain stimulation and spontaneous migraine attacks. In one study, participants with migraine were scanned during a spontaneous attack and compared to a control population revealing a change in connectivity between cognitive-related networks, specifically the Executive Control Network and the Dorsal - Ventral Attention System, as well as abnormal connectivity between the middle frontal gyrus and the insula [10, 11]. Another study, employing positron emission tomography, found consistent activation of the insula and prefrontal cortex, among other areas, during the first 24 h of spontaneous migraine attacks, when compared to the same cohort’s interictal phase [12]. A report exploring attentional networks using task-fMRI under induced pain condition, observed distinct patterns of neural cognitive-pain interaction in migraine compared to controls including deactivation of the dorsolateral prefrontal cortex and left dorsal anterior midcingulate cortex [13]. The reported findings observed effects of pain or spontaneous migraine attack on brain function despite their small samples of 5 to 20 volunteers [10–13]. These studies report on heterogeneous samples, including both cases with and without aura [12], chronic as well as episodic migraine [13], or compared different cohorts of cases in the ictal phase to controls [10, 11].

To the best of our knowledge, the patterns of brain activity while performing cognitive tasks, have not been studied during spontaneous migraine attacks. The aim of our study was to tackle the changes of neural resources subserving working memory during spontaneous migraine attacks and compare them to a retest assessment conducted in pain-free periods within the same cohort. To assess this cognitive domain comprehensively, we also evaluated cognitive performance with a brief neuropsychological battery. Based on previous findings and using a similarly sized sample [10–13], we hypothesized to observe a decline in N-back performance accompanied by activation in pain related areas during the ictal phase when compared to the interictal phase.

## Methods

We conducted a prospective test-retest within-subject design study including neuropsychological evaluation and fMRI acquisition. This study is part of a larger research project that also included perfusion evaluation using arterial spin labelling, which results have been published elsewhere [14]. The study protocol and statistical analysis were not preregistered. The neuroimaging and neuropsychological assessment protocols and patients consent form were reviewed and approved by the Hospital da Luz Ethics Committee.

### Population

Adults diagnosed with episodic migraine without aura according to the International Headache Society criteria [15] (ICHD 2018) were recruited during a medical appointment at the Headache Outpatient Clinic of Hospital da Luz. Additional inclusion criteria required right handedness and absence of prophylactic migraine medication at inclusion. Exclusion criteria consisted in the presence of psychiatric disorders, psychiatric medication or the presence of neurological diseases other than migraine. All participants signed the informed consent.

### Procedure

Clinical and sociodemographic data were collected at the recruitment session through a structured clinical interview by a neurologist, including age of migraine onset and current attack frequency, duration and intensity. Migraine disability was assessed through the headache impact test (HIT-6) [16].

The study protocol consisted in two sessions, the first of which was conducted during a spontaneous migraine attack (ictal session, S-ictal). For this session, patients were instructed to contact the study team by phone at the beginning of an attack if they were experiencing a minimal pain intensity of 4 in a 0–10 of the visual analogue scale (VAS). Once the availability of the scanner was confirmed, patients were invited to the MRI department and instructed to abstain from taking acute migraine medication up to 12 h before the scan. Attack-associated symptoms other than pain were recorded before the exam.

The second session took place at least 1 month after the first session, during an interictal phase (S-interictal), defined as being headache free for at least 48 h before and after the scan session, which was confirmed by a telephone contact 72 h after that session.

In both sessions, a brief neuropsychological evaluation and screening of depressive symptoms with the Beck Depression Inventory (BDI) [17] was performed additionally to the scan. Session order was fixed, ictal first, followed by interictal.

### Neuropsychological battery

We used a neuropsychological battery that had been previously applied to migraine patients. It is focused on attention and executive functions [18], including the following tests: Stroop Test [19] as a measure of inhibitory control; Trail Making Test B for alternate attention and shifting ability; a Phonological Verbal Fluency task (with letters P and M), for verbal initiative and monitoring; Finger Tapping [20] to measure motor speed; Trail Making Test A [21] and Symbol Digit from the Wechsler Adult Intelligence Scale-III (WAIS-III) [22] for sustained attention and visual processing speed.

### Image acquisition

MRI data was obtained on a 3 Tesla Siemens Verio Systems scanner with a 12-channel radiofrequency head coil. In both sessions, a Blood Oxygen Level Dependent (BOLD) fMRI acquisition was performed using a gradient echo-planar imaging (GE-EPI) pulse sequence (TR/TE = 2000/30 ms, voxel size = 4.0 × 4.0 × 3.6 mm^3^, number of slices = 22, number of volumes = 210). Anatomical images were collected using a T1-weighted sequence (Magnetization Prepared Rapid Acquisition Gradient-Echo (MPRAGE) TR/TE = 2250/2.26 ms, voxel size = 1 × 1 × 1 mm^3^).

### N-back task

A block design of the verbal N-back task with 2 conditions (0-back and 2-back) was administered using Nordic NeuroLab hardware and goggles (www.nordicneurolab.com). The N-back paradigm was originally developed by Gevins and Cutillo in 1993 [23] to assess working memory using electrophysiology measures and was subsequently adapted for fMRI a year later [24]. It has been widely used ever since because it requires the updating and manipulating of remembered information, placing different levels of demand on working memory [25]. In the verbal N-back, participants are required to monitor a series of letters shown on the screen and to respond whenever a target letter appears. The 0-back condition was used to access selective attention and vigilance networks, where the target is every appearance of a randomized pre-selected letter in the letter sequence. In the 2-back condition, the target letter is defined as any letter that is identical to the one presented two positions previously in the sequence. By combining both conditions, 0-back as baseline and 2-back for working memory, brain regions related to the active maintenance of information about the stimuli were identified^23^. Each block lasted for 42 s, with a sequence of 21 letters that was presented in a pseudorandomized order for 1 s each, with a 1-s interstimulus interval. Five blocks of conditions 0-back and five of 2-back were presented, with a total duration of 420 s or 7 min. Hit and incorrect response rates are used as behavioural measures of the task.

### Data analysis

The fMRI data was analysed using FSL, the FMRIB Software Library (https://fsl.fmrib.ox.ac.uk/fsl/fslwiki/). Pre-processing consisted of motion correction, fieldmap distortion correction, high-pass temporal filtering (frequency cutoff = 100 s) and spatial smoothing (Gaussian kernel with FWHM = 5 mm). Functional images were registered into the high-resolution anatomical images of the same patient, which were in turn registered to the Montreal Neurological Institute (MNI) standard space using nonlinear registration. First-level statistical analysis was performed using a general linear model (GLM) including two explanatory variables (EVs) generated based on boxcar functions corresponding to the presentation of the 0-back and 2-back stimulus blocks, each convolved with a double-gamma hemodynamic response function. Moreover, standard motion parameters were also included in the GLM as confound EVs. The contrast 2-back > 0-back was defined to assess brain activation associated with working memory. Group analysis of this contrast was performed voxel wise for the whole brain, using the well-established methodology implemented in the FSL tool FEAT/FLAME1 [26], this time building a GLM for repeated measures analysis and testing for the average effect across sessions as well as for differences between sessions [27]. Correction for multiple comparisons was performed using a cluster significance threshold of *p* < 0.05 (and an initial voxel *z* > 2.3) This procedure is consistent with the one used in previous studies [28, 29].

We further investigated whether the activity of the brain region exhibiting differences between phases is related to the neuropsychological and clinical scores of the patients. For this purpose, we defined a region of interest (ROI) based on the cluster exhibiting significant differences between phases (S-ictal > S-interictal) obtained in the whole brain group analysis We then used FSL’s tool Featquery to compute the mean BOLD signal change in this ROI, for each patient and session.

IBM SPSS Statistics 28 version was then used for statistical analysis. Given the sample size (*N* = 11) we used nonparametric measures, and no statistical power calculation was conducted. We applied two-tailed Spearman correlations between disease duration and attack frequency, the seven clinical parameters of the ongoing migraine attack: pain duration in hours and the intensity of pain, nausea, photo and phonophobia, movement intolerance and difficulties in concentration (rated in VAS scale), N-back performance (accuracy and incorrect %) and the scores obtained in the HIT-6 and BDI questionnaires. All the above with the main ROI analysis result using a significance level of *p* < 0.005 applying Bonferroni correction for 14 comparisons. For neuropsychological and N-back test-retest data, the Wilcoxon test for related samples was used, and statistical significance was set at *p* < 0.05 and adjusted for multiple comparisons using the Signed Rank test from SPSS software.

## Results

### Population

Fourteen women with episodic migraine without aura were recruited, and eleven completed the protocol. Two participants did not complete both sessions of the protocol and another participant lacked the behavioural data of the N-back, recorded in the second session, due to technical difficulties. Table 1 provides clinical and sociodemographic data from the final sample of 11 participants. Symptoms at S-ictal are also presented. The HIT-6 questionnaire had a median score of 52, interpreted as experiencing a moderate impact of migraine.

**Table 1:** Demographic and clinical data

	Median	IQR*
Age (years)	32.0	27–44
Education (years)	16.0	12–18
Disease duration (years)	20.0	19–31
Attack frequency (per month)	2.0	1–4
Usual attack duration (hrs.)	24.0	12–48
Usual attack intensity^1^	7.5	6–8
Time between sessions (days)	62.0	54–75
HIT-6 score	52.0	50–55
Studied attack		
Pain duration (hrs.)**	8.2	5.5–22.5
Pain intensity^1^	6.0	6.0–8.0
Nausea^1^	4.0	3.0–5.0
Photophobia^1^	5.0	3.0–5.0
Phonophobia^1^	5.0	3.0–7.0
Worsening with movement^1^	4.0	2.0–6.0
Concentration difficulties^1^	6.0	2.0–7.5

*Interquartile range

^1^VAS scale: 0 (absence of symptom) – 10 (maximum intensity of symptom)

HIT-6: headache impact test

**Pain duration since the studied spontaneous attack started at session-ictal

### Neuropsychological assessment

All neuropsychological tasks showed, on average, a lower performance in S-ictal, the first session, compared to the S-interictal (results presented in Table 2). Subjects were slower in both TMT A and TMT B and had lower scores in cognitive flexibility, processing speed (letter fluency, digit symbol), with a lower number of words named and symbols drawn respectively. This difference was significant in Stroop test’s interference score (*p* = 0.010) and in the finger tapping of the left hand (*p* = 0.006). There was no significant difference in depressive symptoms between sessions (*p* = 0.289). The average BDI score was 7.3, indicating “no depression”, and was higher during S-ictal compared to S-interictal (9.9 vs 4.7 points, *p* = 0.289). Performance on the 2-back task increased from an average hit rate of 87% during the attack, to a 92% in the interictal session, which was not significant.

**Table 2:** Neuropsychological assessment per phase at session (raw data)

	Total	S-ictal	S-interictal	*p* value*
TMT A (seconds; mean (SD))	24.1 (5.1)	25.8 (4.3)	22.4 (5.5)	0.065
TMT A errors (mean (SD))	0.2 (0.4)	0.3 (0.5)	0.2 (0.4)	1.000
TMT B (seconds; mean (SD))	66.5 (19.0)	71.3 (20.4)	61.7 (17.1)	0.227
TMT B errors (mean (SD))	0.6 (0.9)	0.3 (0.7)	0.9 (0.9)	0.219
Stroop Interference (mean (SD))	60.8 (13.4)	55.0 (13.6)	66.6 (10.7)	0.012*
Stroop errors (mean (SD))	0.7 (0.7)	0.9 (0.8)	0.5 (0.5)	0.289
Finger tap dominant (mean (SD))	49.6 (6.8)	50.9 (8.7)	48.3 (4.3)	0.227
Finger tap not dominant (mean (SD))	50.6 (8.2)	44.7 (6.8)	56.5 (4.2)	0.012*
Letter fluency (mean (SD))	15.7 (5.1)	14.9 (5.0)	16.6 (5.4)	0.754
BDI score (mean (SD))	7.3 (9.7)	9.9 (13.1)	4.7 (3.5)	0.289
2N-back accuracy in %	90.2 (9.2)	88.4 (9.2)	92.0 (9.3)	0.344
2N-back incorrect responses in %	3.5 (5.0)	3.6 (5.8)	3.3 (4.3)	1.000

*Related samples Wilcoxon nonparametric test, adjusted for multiple comparisons at significance level *p* < 0.050

Behavioural measures did not correlate with the time between assessments in the 2-back (Spearman *r* = .18; *p* = 0.600), nor the in Finger Tap left (*r* = 0.18; *p* = 0.601) or Stroop (*r* = .09; *p* = 0.790).

### fMRI

**Fig. 1:**
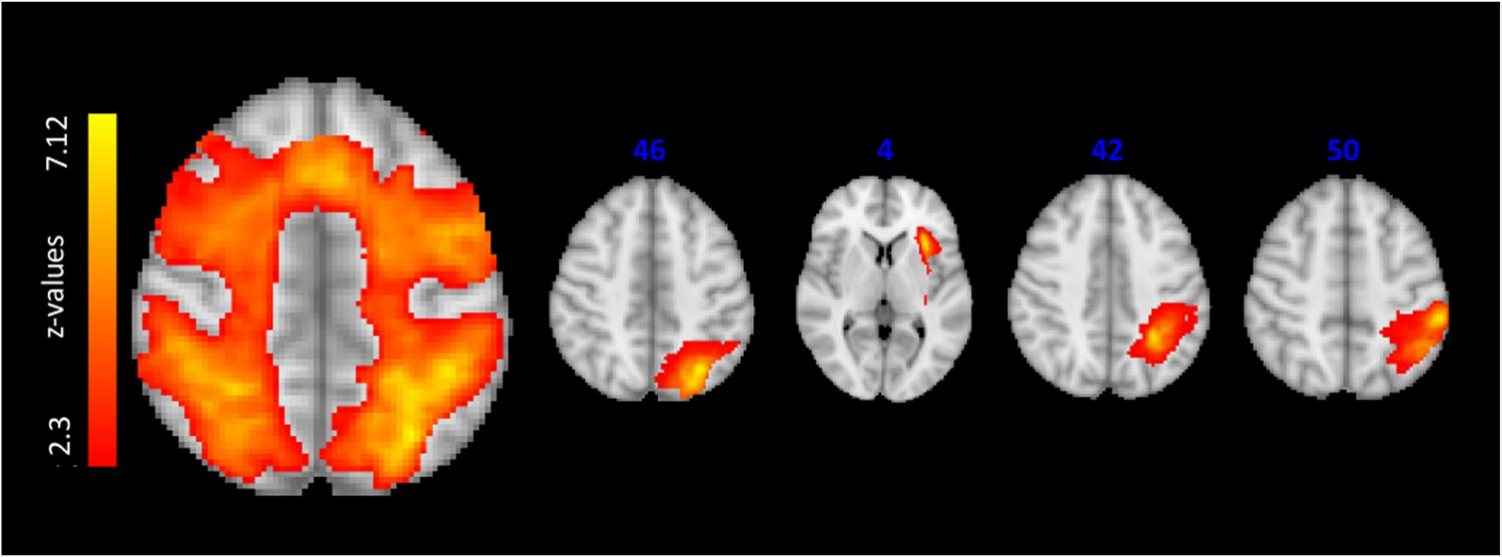
Group mean activation with the N-back task across patients and sessions. *z*-stat map represented in colour overlaid on the MNI template anatomical image. Four axial slices are shown corresponding to the four clusters found

Group mean maps of activation during the N-back (contrast 2-back > 0-back) showed significant activity in areas relevant to verbal working memory, including the lateral occipital cortex, parietal lobule and insula. There was an overall higher activation in the first session (S-ictal) compared to S-interictal (Fig. 1 and Table 3).

**Table 3:** MNI coordinates for each cluster of the group mean activation with the N-back task across patients and sessions. The maximum *z*-stat value and the brain region are identified on the Harvard-Oxford atlas. Cluster significance was thresholded at *p* = 0.05 and *z* > 2.3

MNI [mm]	z-max	Brain regions
− 30 − 66 46	7.05	Lateral occipital cortex left
− 30 20 4	6.80	Insular cortex left
− 26 − 46 42	6.79	Superior parietal lobe left
− 46 − 46 50	6.74	Supramarginal gyrus, angular gyrus left

**Fig. 2:**
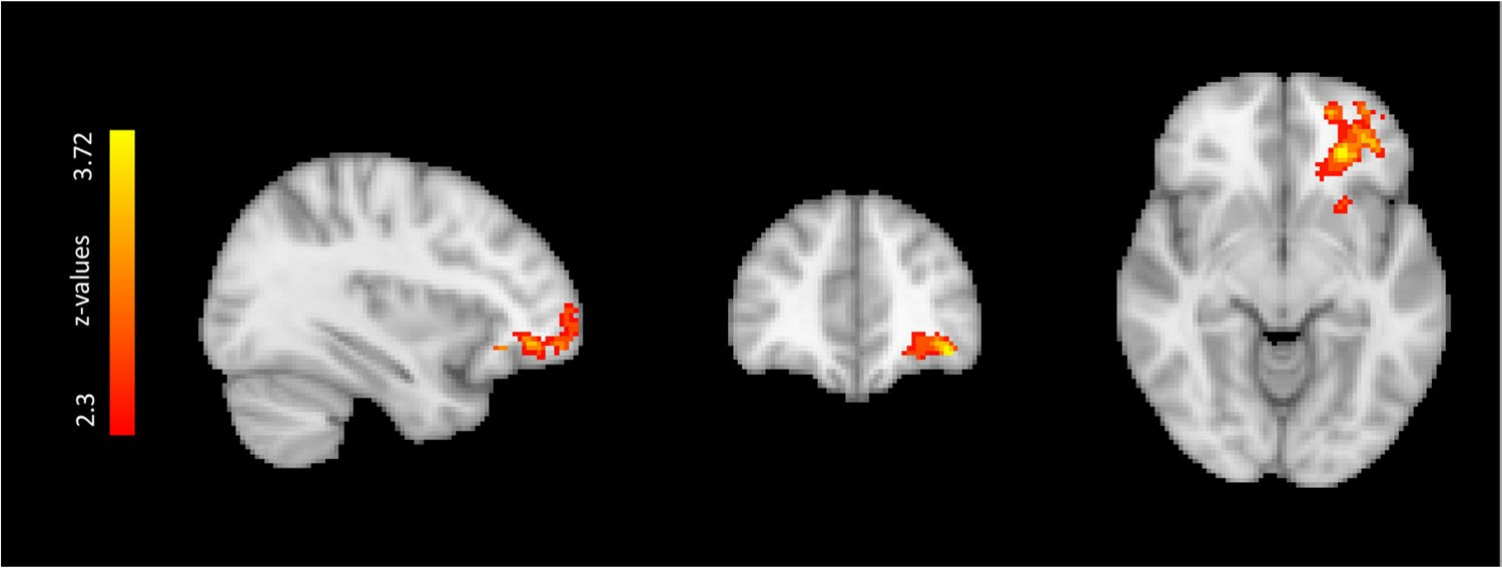
Map of significant brain activation differences between sessions (S-ictal > S-interictal): the *z*-stat map is represented in colour overlaid on the MNI template structural image. Three orthogonal slices are shown in the cluster peak coordinate (− 34 42 − 8)

In the analysis of S-ictal > S-interictal, significantly higher activation was found in the frontal lobe, including regions of the left frontal pole and the orbitofrontal cortex (Fig. 2 and Table 4). No significantly higher activation was found for S-interictal > S-ictal. This cluster of activation was subsequently utilized as ROI to compare its level of activation for each patient and session with different clinical and neuropsychological variables.

**Table 4:** Cluster properties of significant brain activation differences between sessions (S-ictal > S-interictal): The maximum *z*-stat value and the brain region identified on the Harvard-Oxford atlas namely the left frontal pole and orbital cortex. Cluster significance was thresholded at *p* = 0.05 and *z* > 2.3

MNI [mm]	*z*-max	Brain regions	Cluster *p* value	Cluster size
− 34 42 − 8	3.42	Frontal pole, orbital cortex	0.0106	805

**Fig. 3:**
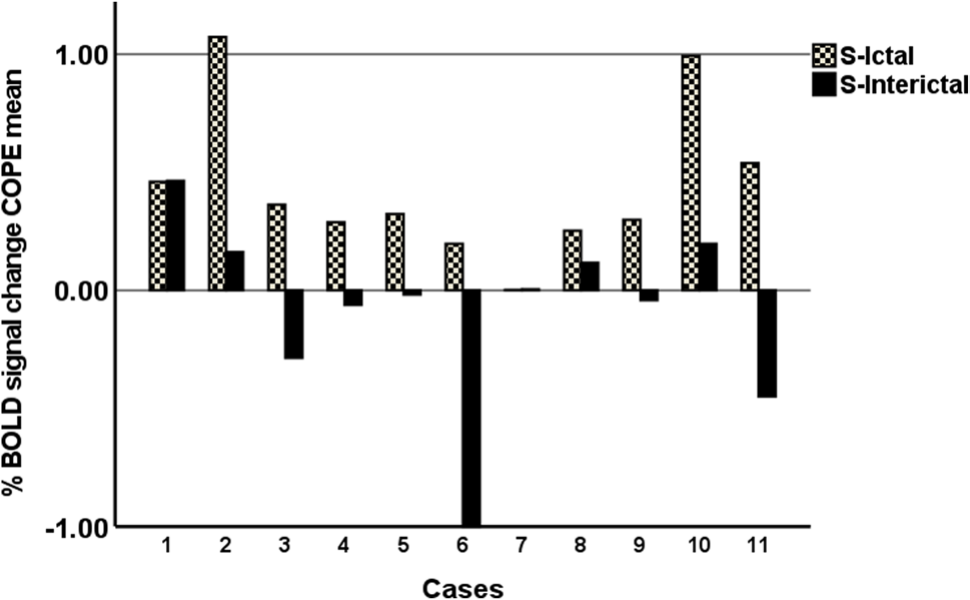
Percentage of BOLD signal change in the prefrontal ROI corresponding to the S-ictal > S-interictal significant differences, for each subject and session. The BOLD signal change is greater in the ictal phase compared with the interictal phase for all patients except two (N° 1 and 7)

The average percentage of BOLD signal change during N-back in the ROI is shown for each session and patient in Fig. 3. Consistently with the group results, most patients showed activity in this ROI during S-ictal, which was diminished or even deactivated during S-interictal (Fig. 3).

The activation of this ROI in the left frontal pole and orbitofrontal cortex during the migraine attack did not correlate with the N-back performance in accuracy (*r* = − .34; *p* = 0.304) nor incorrect responses (*r* = .50; *p* = 0.115). It neither correlated with the BDI questionnaires (r = .324; *p* = 0.331) nor the HIT-6 scores where only a trend was observed (*r* = .71; *p* = 0.014). Neither did we find significant correlations between the percentage BOLD signal change in the ROI and clinical features of disease duration (*r* = .17; *p* = 0.619), attack frequency (*r* = .10; *p* = 0.779), nor with the studied migraine attack clinical parameters, namely: pain duration (*r* = − .25; *p* = 0.467), pain severity (*r* = − .28; *p* = 0.412), nausea (*r* = − .36; *p* = 0.275), photophobia (*r* = − .33; *p* = 0.322), phonophobia (*r* = .01; *p* = 0.989), worsening with movement (*r* = − .27; *p* = 0.414) and concentration difficulties (*r* = 0.11; *p* = 0.748).

## Discussion

In a prospective within-subject test-retest study, we investigated working memory in migraine using neuropsychological assessment and a cognitive task with fMRI, during and between spontaneous migraine attacks. We found that patients obtained comparatively lower cognitive scores during migraine attacks and also that performance in a working memory task during the attack increased the activation of areas related to inhibitory control and pain. To our knowledge, this is the first study documenting the neural correlates of a demanding executive task performed during migraine attacks, a finding that contributes to explain patients’ complaints.

### Neuropsychological assessment

Cognitive scores of both sessions were within expected ranges based on age and education. However, an overall enhancement in neuropsychological performance was evident during the second, S-interictal assessment, compared to S-ictal, which was significant in the Stroop test and non-dominant finger tapping. A comparable trend of improvement has been noted in a test-retest study using the same battery in healthy controls and interictal migraine participants, showing a significant difference only in the Stroop test^17^. These observed improvements may be attributed to the learning effect on the second session. While the time interval between evaluations was longer in the earlier study (80 days), the absolute enhancement observed here surpassed what was observed in the previous data. This implies that the absence of pain could have influenced the magnitude of the expected learning effect, in our sample.

### fMRI

The improvement in the N-back task performance and the reduced brain activity that were observed in the second session, although not correlated, could be explained either by a learning effect or a decline in mental effort, associated to the absence of pain. A study using a 2-back task in healthy individuals with a shorter test-retest interval (14.6 days), observed a decrease in functional activity in the parietal and dorsolateral prefrontal cortex between first and second session, which was attributed to the learning effect [30]. The observed improvement in performance that increased from 93.2 to 95.5% of hit accuracy in that study was not statistically significant and less expressive than what was found in our data. The higher magnitude of difference observed in our participants suggests that there was an additional effect of the migraine attack affecting participants’ performance in the first session. This finding supports the hypothesis that pain impairs working memory function, as suggested by a report of a negative relationship between pain severity and N-back behavioural measures in subjects with diverse pain conditions [31]. Altogether, this data indicates the cost of sharing limited attentional resources between cognitive functions and pain.

In fact, we identified a cluster on the left orbital prefrontal cortex that was significantly more activated in the ictal session during the N-back paradigm. This area is not generally considered to be part of the working memory network, regardless of the modality (auditory or visual) in which the cognitive effort is generated [32]. For verbal working memory, namely with the letter N-back, areas activated include the middle frontal gyrus and parietal lobe regions [33]. Conversely, the orbito-prefrontal cortex is relevant for inhibition, impulse control and decision-making [34]. However, it is also observed under trigeminal pain stimulation and ictal phase, along with activation of somatosensory cortices and other brain areas indicating that it can be related to pain processing [35]. Earlier research has also reported high functional connectivity between regions associated with pain and sensory processing, such as the right thalamus and contralateral pain processing regions, including the orbitofrontal cortex [36]. In a similar study which also compared spontaneous attacks and interictal phase in migraine without aura but not during a cognitive task, significant functional connectivity between the insula and the frontal pole was noted during painful stimulation in patients with high frequency of attacks [37].

Alternatively, the higher activation of the left orbital prefrontal cortex observed in the ictal phase could suggest that patients are employing greater inhibitory control to perform the task.

No significant correlation was identified between the prefrontal cortex activation during S-ictal and N-back performance, depression questionnaire, disease duration, attack frequency or the clinical features of the studied attack. However, individuals reporting higher disability (according to the HIT-6) displayed higher brain activity in that region during the attack, although statistical significance was not achieved. A preceding study investigating resting state in interictal migraine without aura established an inverse correlation between the HIT-6 score and impaired connectivity in the somatosensory cortex [38].

Taken together, these findings reinforce the concept that activity in brain regions associated to pain processing might influence the neural correlates of cognitive performance during migraine attacks.

### Limitations

In an ideal study design, the migraine phases would be balanced, and session repetition should be randomized to minimize the learning bias. However, for practical recruitment issues and to improve effective participation, the researchers prioritized ictal sessions. A similar design was used in a study which reported difficulties alternating the sessions while studying brain activity during spontaneous migraine attacks [39]. Having a healthy control group would be necessary to compare the test-retest learning and familiarity effect of the task and the context of the fMRI exam. The sample size of eleven exclusively female participants limits the generalizability of the findings. Another relevant limitation is that the scans were made in different time frames within the attack, and there is evidence that brain activation may differ according to the timing from the onset [40].

## Conclusion

The present study used fMRI to investigate the neural basis of working memory during spontaneous migraine attacks. Our results showed a decrease in performance during ictal phase compared to interictal, although scores of both sessions were within expected ranges based on age and education. We also observed significant prefrontal activation during the ictal phase indicating a potential interaction between the pain network and areas involved in cognitive processing. While further research is necessary to fully understand the impact of migraine attacks on cognitive functioning, our findings support the existence of a neural basis for cognitive complaints reported by migraine patients during attacks.

## Data Availability

The data that supports the findings of this study are available from the authors upon reasonable request.
